# Risk factors of metatarsal stress fracture associated with repetitive sports activities: a systematic review

**DOI:** 10.3389/fbioe.2024.1435807

**Published:** 2024-08-08

**Authors:** Jiayi Sun, Chenglong Feng, Yaming Liu, Mianjia Shan, Zilin Wang, Weijie Fu, Wenxin Niu

**Affiliations:** ^1^ Key Laboratory of Exercise and Health Sciences, Ministry of Education, Shanghai University of Sport, Shanghai, China; ^2^ Translational Research Center, Shanghai Yangzhi Rehabilitation Hospital, School of Medicine, Tongji University, Shanghai, China

**Keywords:** sports injury, metatarsal stress fracture, risk factors, anatomical characteristic, biomechanical effect

## Abstract

**Background:**

Metatarsal stress fracture is common in people engaged in repetitive weight-bearing activities, especially athletes and recruits. Identifying risk factors in these contexts is crucial for effective prevention.

**Methods:**

A systematic search on Web of Science, PubMed, EBSCO, SPORTDiscus, MEDLINE, and Cochrane Library was conducted and the date range for the retrieval was set from January 1984 to April 2024.

**Results:**

32 eligible studies were selected from 1,728 related research. Anatomical and biomechanical factors, such as higher foot arch, abnormal inversion/eversion of foot, and longer metatarsal length or larger angles, relatively influence stress fracture risk. However, given that there is no standardized measurement, the results remain to be examined. Soccer is associated with fifth metatarsal fractures, while long-distance running and recruit training often lead to fractures of the second or third metatarsals. High exercise intensity, non-adaptive training, and inadequate equipment heighten fracture risk.

**Conclusion:**

This review highlights the complex interplay of anatomical, biomechanical, and sports-related factors in the risk of metatarsal stress fractures. Relatively, high arches, specific metatarsal morphologies, and foot inversion/eversion patterns are significant risk factors, particularly among athletes. Sports type also correlates with metatarsal stress fracture locations. Despite extensive research, study heterogeneity and inherent biases necessitate cautious interpretation. Comprehensive, multifactorial approaches and personalized injury prevention strategies are essential for reducing the incidence of these injuries and improving the health and performance of athletes.

## 1 Introduction

Metatarsal stress fractures, among the most common types of stress injuries affecting the lower limb bones, can manifest as either partial or complete fractures. They are typically caused by the repetitive application of stress over time, leading to cumulative damage and abnormal bone remodelling processes. Most running-related injuries are caused by these so-called stress or fatigue fractures.

The high-risk population of metatarsal stress fracture is mainly athletes and recruits, accounting for nearly 75% of all stress fractures in athletes (Glasoe et al., 2002; Welck et al., 2017). Due to the unique anatomical characteristics and the differing mechanical environments of the metatarsals, certain bones are more prone to stress fractures. For instance, during recruit training, the second and third metatarsal are particularly susceptible to stress fracture, commonly known as “March fracture” (Ramponi et al., 2017; Fujitaka et al., 2020). Additionally, the fifth metatarsal stress fracture is more prevalent in elite athletes such as soccer players and runners. These fractures are frequently challenging to treat because of the lower blood supply of the proximal diaphyseal, which will increase the risk of delayed union or non-union (Karnovsky et al., 2019; Kizaki et al., 2019; Fujitaka et al., 2020).

Repetitive sports activities, which involve consistent and high-impact movements, significantly contribute to the risk of developing metatarsal stress fractures. Such activities place continuous strain on the bones, leading to microdamage that can accumulate over time if not properly managed (Rice et al., 2019). Identifying the specific risk factors associated with these activities is crucial for developing effective prevention and treatment strategies.

Given these challenges, it is essential to clarify the influencing factors on metatarsal stress fracture for prevention and protection management. Current research discusses various risk factors for metatarsal stress fractures, including anatomical, biological, and biomechanical features, but often lacks depth and context-specific analysis. Different theories exist about the pathomechanics of these fractures, but the pathogenesis remains controversial, and the complex interplay of factors in repetitive sports activities is often overlooked.

This review aims to systematically review and analyses previous research to provide a comprehensive understanding of the risk factors for metatarsal stress fractures in repetitive sports activities. These findings will help explore the mechanisms of stress fractures and inform intervention strategies for trainers and physicians.

## 2 Methods

### 2.1 Literature search strategy

This article conducted a systematic review of the published studies in peer-reviewed journals related to the risk factors of metatarsal stress fracture associated with repetitive sports activities and was written in accordance with Preferred Reporting Items for Systematic Review and Meta-Analyses (PRISMA) guidelines (Page et al., 2021). The databases Web of Science, PubMed, EBSCO, SPORTDiscus, MEDLINE, and Cochrane Library were thoroughly searched using a combination of literature search terms similar to the one displayed in Table 1 for PubMed. Furthermore, to find other pertinent literature, the references of the included literature were tracked down. The date range for the retrieval was set from January 1984 to April 2024.

**TABLE 1:** Search terms used in PubMed.

Database	Search terms	Filters
PubMed	((“Running” [Mesh] OR “Long-distance runn*” [tiab] OR “Marathon running” [Mesh] OR “Marathon runn*” [tiab] OR “Soccer” [Mesh] OR “Basketball” [Mesh] OR “Volleyball” [Mesh] OR “Jumping” [Mesh] OR “Jump*” [tiab] OR “High-impact exercise” [Mesh] OR “High-impact exercise” [tiab] OR “Aerobic exercise” [Mesh] OR “Aerobic exercise” [tiab] OR “Repetitive Exercise” [Mesh] OR “Repetitive Exercise” [tiab] OR “Repetitive Movement” [Mesh] OR “Repetitive Movement” [tiab])) AND ((“Metatarsal Bones” [Mesh] OR “Metatarsal Fractures” [Mesh] OR “Metatarsal Fractures” [tiab] OR “Metatarsal Stress Fractures” [tiab] OR “Stress Fractures”[Mesh] OR “Stress Fracture”[tiab] OR “March Fracture” [tiab] OR “Jones Fracture" [tiab])) AND ((“Risk Factors” [Mesh] OR “determinant” [tiab] OR “determinants” [tiab] OR “risk” [tiab] OR “risks” [tiab] OR “etiology” [tiab] OR “Risk” [Mesh:NoExp] OR “etiology” [sh:NoExp] OR Etiology/Narrow [filter])) AND (“Cohort Studies” [Mesh] OR “Case-Control Studies” [Mesh] OR “Cross-Sectional Studies” [Mesh] OR “Longitudinal Studies” [Mesh])	Humans, English, 1984–2024

### 2.2 Data collection and processing

The following screening circumstances were subjected to both automatic and manual screening. The inclusion criteria for this systematic review on risk factors of metatarsal stress fractures associated with repetitive sports activities were as follows: 1) original research articles (RCTs, cohort, case-control, and cross-sectional studies) involving participants engaged in repetitive sports activities (e.g., runners, soccer players). 2) Studies needed to report risk factors such as anatomical or biomechanical factors, training type, frequency, shoe type, surface hardness, and include clear diagnostic criteria for metatarsal stress fractures.

Exclusion criteria were: review articles, commentaries, opinion pieces, conference abstracts, unpublished theses, animal studies, studies on populations not engaged in repetitive sports activities, articles without specific data on risk factors or metatarsal stress fractures, and studies that did not provide clear diagnostic criteria for metatarsal stress fractures.

### 2.3 Risk of bias assessment

All risk factor studies were assessed for risk of bias (RoB) by two reviewers independently using the Quality in Prognostic Studies (QUIPS) tool (Hayden et al., 2013). For risk model studies, RoB was determined using the Prediction model Risk Of Bias Assessment Tool (PROBAST) (Moons et al., 2019). Disagreement was resolved by consensus. A third reviewer made the final decision in cases where no consensus could be reached.

### 2.4 Literature quality assessment

Studies that were included following the selection processes underwent a process of critical appraisal by two reviewers to assess their methodological quality.

The Critical Appraisal Skills Program (CASP) toolkit (“CASP Checklists - Critical Appraisal Skills Programme”) was used to assess and appraise cohort, case-control, and controlled studies (Critical Appraisal Skills Programme, 1993), whereas the AXIS tool was used to appraise cross-sectional studies (Downes et al., 2016). The CASP cohort study scale consists of 12 questions and the CASP case-control study checklist consists of 11 questions addressing the quality, validity, and relevance of screening and study design. The AXIS evaluation questionnaire contains a checklist of 20 questions, which includes 11 questions assessing research objectives and methods, 7 questions related to research findings and discussion, and 2 questions related to ethics.

### 2.5 Data synthesis

We summarized the findings in tables, figures, and text, focusing on the main risk factors for metatarsal stress fractures. The analysis categorized risk factors into three primary groups based on anatomical, biomechanical, and sports-related characteristics. A meta-analysis could not be performed due to clinical heterogeneity with respect to population and definition of outcome(s).

## 3 Results

### 3.1 Retrieval results

A total of 1,728 articles were retrieved, and in addition, 16 articles were supplemented by tracing the reference lists of the related articles. After eliminating non-English, review, and conference articles, 1,698 articles remained. A total of 1,515 duplicate articles were excluded by automatic retrieval. Then 133 articles were excluded according to the title and abstract, and 50 articles were screened for full text reading. After full text reading, a total of 32 articles associated with risk factors of metatarsal stress fracture were finally screened.

**FIGURE 1:**
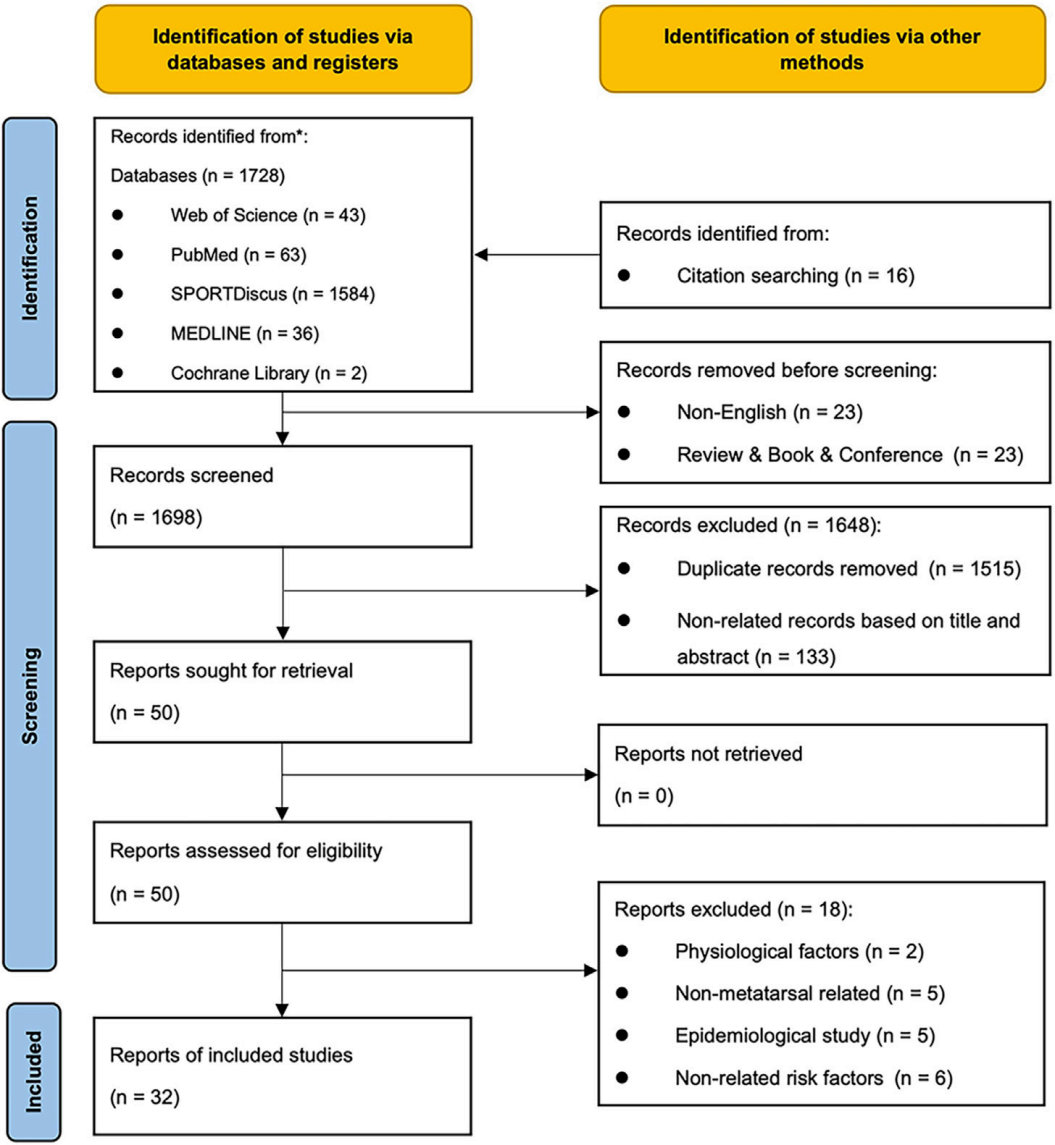
PRISMA 2020 flow diagram.

The specific search and screening process are all presented in the PRISMA flow chart shown in Figure 1 (Page et al., 2021).

### 3.2 RoB assessment

**FIGURE 2:**
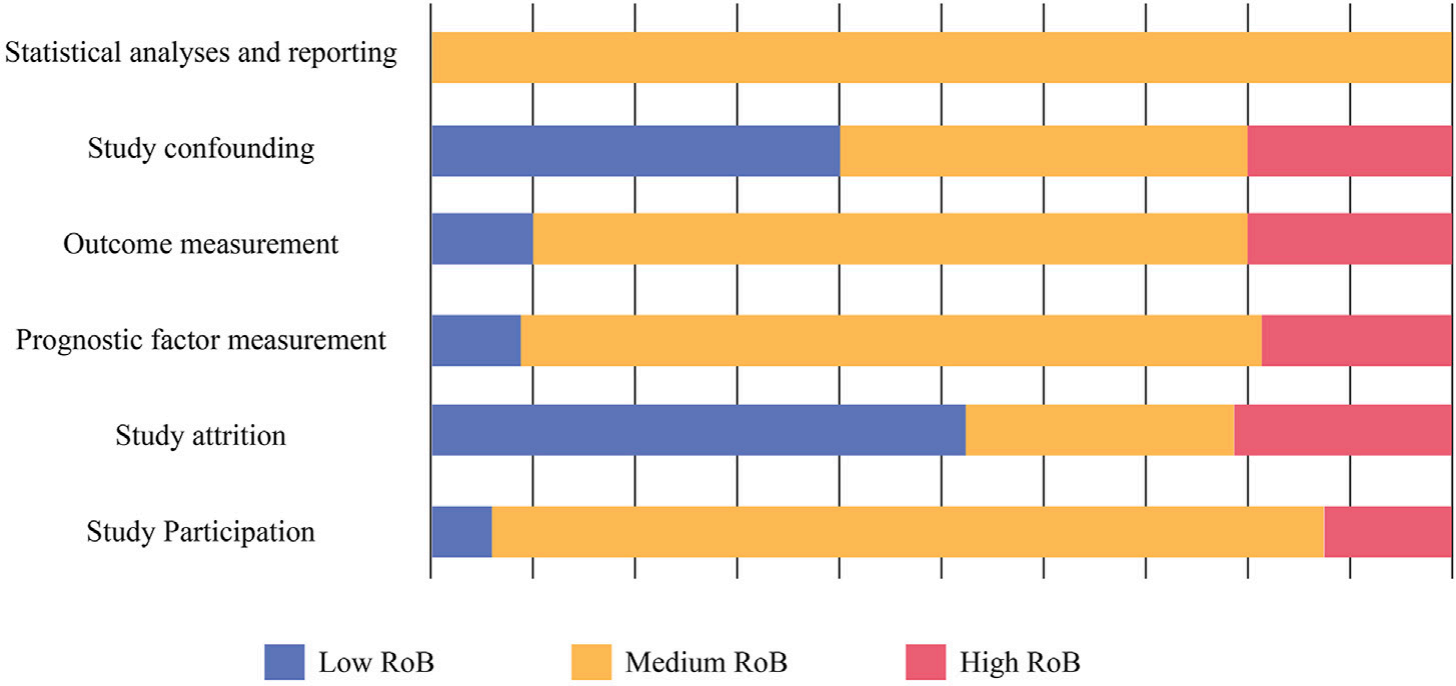
Risk of bias (RoB).

The RoB in the domains “study confounding” and “study attrition” was low. These domains showed the highest RoB, mainly due to insufficient reporting (Table 2; Figure 2).

**TABLE 2:** Rating for individual studies.

References	Study participation	Study attrition	Prognostic factor measurement	Outcome measurement	Study confounding	Statistical analyses and reporting
Fujitaka et al. (2020)	M	L	M	H	M	M
Hotfiel et al. (2020)	H	L	M	M	L	M
Lv et al. (2020)	L	L	M	M	L	M
Karnovsky et al. (2019)	M	L	M	H	H	M
Miyamori et al. (2019)	M	H	M	M	L	M
Miller et al. (2019)	M	L	L	M	L	M
Kizaki et al. (2019)	M	L	M	M	L	M
Saita et al. (2018)	M	M	M	M	H	M
Taylor et al. (2018)	M	L	M	M	M	M
Azevedo et al. (2017)	H	L	M	M	M	M
Sun et al. (2017)	M	L	M	M	H	M
Matsuda et al. (2017)	H	H	H	M	M	M
Carl et al. (2014)	L	M	M	M	L	M
Ekstrand and Van Dijk (2013)	M	H	M	M	M	M
Ekstrand and Torstveit((2012)	M	H	M	M	L	M
Lee et al. (2011)	M	L	M	M	L	M
Hetsroni et al. (2010)	M	M	M	H	H	M
O’Malley et al. (2016)	M	L	M	M	M	M
Dixon et al. (2019)	M	M	M	H	M	M
Rice et al. (2019)	M	M	M	M	H	M
Pihlajamäki et al. (2019)	M	L	M	L	L	M
Shaffer et al. (2006)	M	M	L	M	L	M
Kliethermes et al. (2021)	M	H	H	H	H	M
Bergstra et al. (2015)	M	L	H	M	M	M
Wellenkotter et al. (2014)	M	L	M	M	L	M
Tenforde et al. (2013)	H	H	M	M	M	M
Bischof et al. (2010)	M	L	H	H	M	M
Stolwijk et al. (2010)	M	H	H	M	M	M
Nagel et al. (2008)	M	M	M	M	M	M
Weist et al. (2004)	M	M	H	M	M	M
Bennell et al. (1996)	M	L	M	M	L	M
Sullivan et al. (1984)	M	L	M	M	L	M

Abbreviations: H = high risk of bias; L = low risk of bias; M = medium risk of bias.

### 3.3 Literature content

**FIGURE 3:**
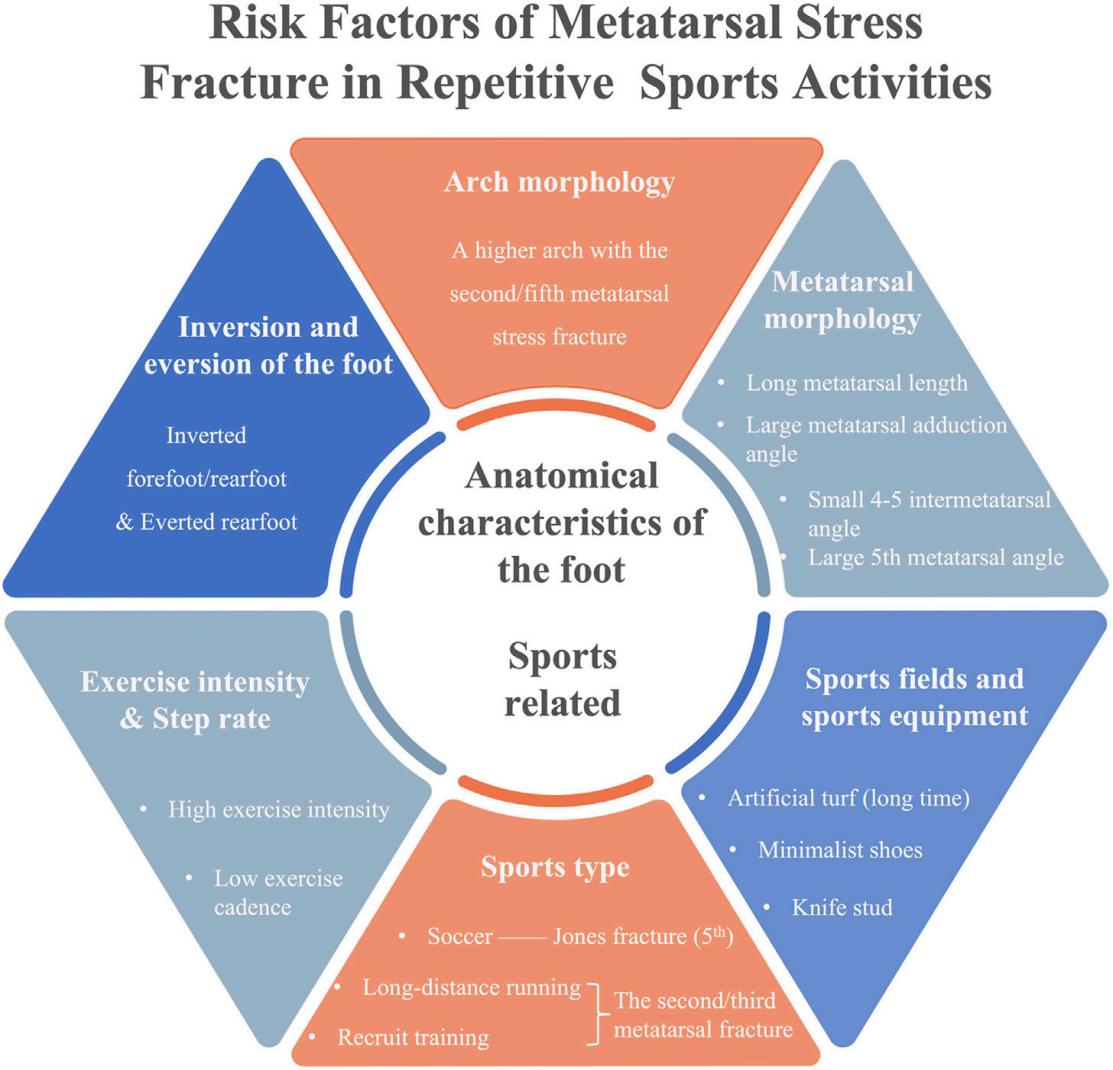
Risk factors of metatarsal stress fracture.

A total of 32 studies were included, including 20 case-control studies and cohort studies, 10 clinical controlled experimental studies, and two cross-sectional study. Relevant demographic information and outcomes regarding the literature were presented in Supplementary Table S2. And the risk factors associated with metatarsal stress fracture were extracted to Table 3 and presented in Figure 3.

**TABLE 3:** Risk factors of metatarsal stress fracture in repetitive sports activities.

Variables			Number of studies
Anatomical characteristics of the foot	Arch morphology		5
	Inversion and eversion of the foot		4
	Metatarsal morphology	Metatarsal length	2
		Metatarsal angles	4
Sports related	Sports type	Soccer	17
		Long-distance running	10
		Recruit training	4
	Exercise intensity and Step rate		15
	Sports fields and sports equipment		6
Other factors (Age, Gender)			7

In the overall quality of literature assessment results, the high-quality research provides strong evidence to support the conclusion of review. However, some studies have potential bias and methodological limitations, which may affect the credibility of the conclusions of the review. Through the quality assessment of literature, 10 high-quality literature and 22 medium-quality literature were presented.

#### 3.3.1 Anatomical environment of the foot

##### 3.3.1.1 Arch morphology

A total of five articles have explored the potential role of arch morphology as a risk factor, but in the review of these relationship, a nuanced picture emerges from the studies reviewed. Three studies suggest that high-arched feet may be associated with an increased risk of metatarsal stress fractures, with specific numerical values providing a clearer definition of what constitutes a high arch. (Lee et al., 2011; Dixon et al., 2019; Fujitaka et al., 2020). For instance, Lee et al. (2011) found that the calcaneal pitch angle (CP) was significantly higher in the fracture group, averaging 27.4°, compared to the non-fracture group, which averaged 18.3°. A higher CP angle may be indicative of a high arch and potentially linked to an increased risk of stress fractures. Furthermore, Dixon et al. (2019) provided data on the arch index, with the non-injured group having an average arch index of 21.97%, while the group with second metatarsal stress fractures had a significantly lower average arch index of 17.74%. The relative risk reduction (RRR) for second metatarsal stress fractures associated with a high arch index was 0.75, with a 95% confidence interval of 0.63–0.89, further supporting the notion that a lower arch index, indicative of a high arch, may be associated with an increased risk of metatarsal stress fractures.

While the other two studies report no statistically significant differences in foot arch measurements between affected individuals and controls (Hetsroni et al., 2010; Matsuda et al., 2017). Matsuda et al. (2017) reported no statistically significant differences in arch height, measured by the Arch Ratio, between the fracture group (17.3% ± 2.2%) and the control group (17.0% ± 2.3%), with a *P*-value of 0.84, indicating that this particular measure may not be a reliable indicator of high arches in relation to stress fractures.

Different studies have used different measurements, including radiologic assessment, plantar pressure measurements, and anthropometric techniques. These results indicate that high-arched feet may be associated with an increased risk, but the specific mechanisms and influencing factors may vary depending on individual differences and measurement methods.

##### 3.3.1.2 Inversion and eversion of the foot

Four studies have indicated a relationship between foot inversion and eversion and the occurrence of the fifth metatarsal stress fracture by using a case-control design, radiographic measurements, and logistic regression analysis, especially among soccer players (Lee et al., 2011; Matsuda et al., 2017; Saita et al., 2018; Kizaki et al., 2019). In addition, Saita et al. (2018) proposed that range limitation in hip internal rotation would lead to subtalar supination and increase the lateral plantar pressure, which would increase the risk of fracture by a factor of 3.03. Kizaki et al. (2019) also provided a risk evaluation index that the ratio of stress fractures in athletes with a greater medial malleolar slip angle (MMSA) was 1.27. These findings could help to identify high-risk individuals and may provide a basis for preventive strategy development.

##### 3.3.1.3 Metatarsal morphology

A total of five articles explored whether metatarsal morphology (length and angle) could be an exact indicator of risk factors. A longer length of the fifth metatarsal was identified as one of the risk factors of the fifth metatarsal stress fracture (Karnovsky et al., 2019; Fujitaka et al., 2020). Although specific thresholds are not specified in the literature, they do provide a reference for follow-up research. Karnovsky et al. (2019) found that the average length of the fifth metatarsal in the fracture group was 90.0 mm, compared to 86.1 mm in the non-fracture group, as observed in a lateral view. Similarly, Lee et al. (2011) reported that the average fifth metatarsal length was longer in the fracture group, with an average of 75.2 mm for feet with Jones fractures and 74.8 mm for feet without Jones fractures, compared to 69.2 mm and 69.5 mm in the non-fracture group for the dominant and non-dominant feet, respectively. These studies mainly focused on the relative relationship of metatarsal length to other anatomical characteristics (e.g., metatarsal angle, foot morphology, etc.) and how these characteristics may be associated with the risk of metatarsal stress fractures.

A large metatarsal adduction angle, a smaller fourth to fifth intermetatarsal angle and a large fifth metatarsal angle were regarded to be associated with the fifth metatarsal stress fracture by using radiographic measurement (Lee et al., 2011; O’Malley et al., 2016; Dixon et al., 2019; Karnovsky et al., 2019). Karnovsky et al. (2019) indicated that the fifth metatarsal angel in the fracture group averaging 3.9° and the non-fracture group averaging 2.6°. Furthermore, Lee et al. (2011) found that the fifth metatarsal lateral deviation angle (MT5-LD) was significantly greater in the fracture group, averaging 5.9°, compared to the non-fracture group’s average of 2.6°.

However, it is important to note that studies such as Karnovsky et al. (2019) and Dixon et al. (2019) have high biases in outcome measurement, which could affect the reliability of these findings.

#### 3.3.2 Sports related

##### 3.3.2.1 Sports type

Repetitive sports activities associated with metatarsal stress fractures can be broadly categorized into three groups: soccer and basketball (17 articles), recruit training (4 articles), and running (10 articles). Soccer is typically associated with fifth metatarsal fractures, while the other two sports typically induce stress fractures of the second or third metatarsals.

Fifth metatarsal stress fractures (MT5 SF) are prevalent in soccer players, attributed to factors like foot anatomy, repetitive stress, rapid load changes, surface and footwear variations, fatigue, hindfoot varus, and metatarsus adductus (Hetsroni et al., 2010; Carl et al., 2014; Clutton and Perera, 2016; Shimasaki et al., 2016; Wamelink et al., 2016). These fractures often occur during preseason due to sudden load changes after rest periods (Ekstrand and Van Dijk, 2013).

In long-distance running, stress fractures account for 5%–16% of injuries, with high incidence among college runners and ultramarathon participants (Hoffman and Krishnan, 2014). The rise in running popularity correlates with increased running-related injuries (RRIs), often due to repetitive stress or overuse (Bischof et al., 2010; Stolwijk et al., 2010; Tenforde et al., 2013). Gradual mileage increases are recommended to mitigate these risks.

##### 3.3.2.2 Exercise intensity and step rate

Twelve studies examine high-intensity movement and adaptation to risk variables for metatarsal stress fracture, respectively (Sullivan et al., 1984; Bennell et al., 1996; Shaffer et al., 2006; Nagel et al., 2008; Bischof et al., 2010; Stolwijk et al., 2010; Ekstrand and Van Dijk, 2013; Tenforde et al., 2013; Matsuda et al., 2017; Miller et al., 2019; Pihlajamäki et al., 2019; Rice et al., 2019). Regular exercise before training also can reduce the risk of fracture during the recruit training (Shaffer et al., 2006; Pihlajamäki et al., 2019).

Additionally, low step rate was identified to be significantly related to metatarsal stress fracture (Wellenkotter et al., 2014; Kliethermes et al., 2021).

##### 3.3.2.3 Sports fields and sports equipment

Different fixation method of stud would indirectly affect the risk of stress fracture by changing the mechanical environment of the forefoot (Sun et al., 2017; Taylor et al., 2018; Lv et al., 2020). Too fast transition from traditional shoes to minimalist shoes was found to be more likely to cause metatarsal stress fracture (Bergstra et al., 2015). Additionally, playing on artificial turf for a long time would make football players more likely to suffer metatarsal stress fracture in comparison to the clay ground (Miyamori et al., 2019).

#### 3.3.3 Other factors

A total of five studies discussed the correlation of young age as one of the risk factors in young football players (under 16 or 17) who exhibit asymmetric plantar pressure and are more prone to Jones fractures compared to adults, likely due to the transition from lower levels of play to professional intensity (Ekstrand and Torstveit, 2012; Azevedo et al., 2017; Rice et al., 2019; Hotfiel et al., 2020). Additionally, women are more susceptible to metatarsal stress fractures (Bennell et al., 1996; Tenforde et al., 2013).

## 4 Discussion

This systematic review synthesizes current knowledge on the risk factors for metatarsal stress fractures associated with repetitive sports activities. Our analysis reveals that anatomical factors, such as arch morphology and metatarsal geometry, and sports-related factors, including specific sports types and training intensities, relatively play significant roles in the development of these fractures. Additionally, foot inversion and eversion, as well as age, have been identified as contributing factors. Despite the extensive research, variability in study design and bias risk necessitates cautious interpretation of these findings. Future studies should aim to standardize methodologies and explore the interactions between multiple risk factors to enhance our understanding and prevention of metatarsal stress fractures.

### 4.1 Anatomical environment of the foot

#### 4.1.1 Arch morphology

Arch morphology is frequently considered an important risk factor for metatarsal stress fractures due to its influence on load distribution across the foot. The evidence supports that a high arch increases the load on the lateral side of the foot in soccer players, contributing to fifth metatarsal fractures. Conversely, in recruits, low arches tend to increase the risk of fractures in the second and third metatarsals due to different loading patterns. These findings highlight the nuanced role of arch morphology in different populations and activities. However, the evidence is mixed regarding the role of foot arch height in the development of metatarsal stress fractures.

From a bias perspective, studies such as Fujitaka et al. (2020) and Lee et al. (2011) have moderate biases in study participation and prognostic factor measurement but high biases in outcome measurement, which might affect the strength of their conclusions. Hetsroni et al. (2010) has a high bias in outcome measurement and study confounding, possibly explaining the lack of significant findings.

In summary, the evidence is mixed regarding the role of foot arch height in the development of metatarsal stress fractures. While some studies point towards a higher arch as a potential risk factor, others do not support this association. This discrepancy may be attributed to differences in measurement techniques, study populations, or the complex interplay between static and dynamic foot characteristics. Further research is needed to standard the approach to assess foot arch and clarify these relationships and to determine whether a higher arch is indeed a risk factor for metatarsal stress fractures. Moreover, the interaction between foot type, footwear, and injury prevention strategies needs to be taken into account.

#### 4.1.2 Inversion and eversion of the foot

Biomechanically, abnormal inversion or eversion alters the distribution of forces across the foot and have been implicated in metatarsal stress fractures, particularly in athletes. Our review identifies that an inverted forefoot, as well as everted and inverted rearfoot, are associated with increased fracture risk, especially among soccer players (Lee et al., 2011; Matsuda et al., 2017; Saita et al., 2018; Kizaki et al., 2019).

Saita et al. (2018) identified limited hip internal rotation as a contributor to abnormal foot mechanics and increased fracture risk. These findings emphasize the importance of comprehensive biomechanical assessments in at-risk populations. However, studies such as Saita et al. (2018) and Kizaki et al. (2019) have moderate biases in study attrition and outcome measurement, indicating a need for cautious interpretation.

The findings suggest that screening for abnormal inversion and eversion patterns should be an integral part of injury prevention programs for athletes. The current evidence is primarily based on small cohort studies, which may limit the robustness of the conclusions. Future research should focus on larger-scale studies and longitudinal designs to better understand the long-term implications of inversion and eversion on fracture risk. Additionally, examining the interaction between foot mechanics and other factors, such as footwear and surface type, could yield valuable insights.

#### 4.1.3 Metatarsal morphology

Metatarsal length and angles have been identified as potential indicators of fracture risk. Our review shows that a longer fifth metatarsal and certain metatarsal angles are associated with increased fracture risk (Lee et al., 2011; O’Malley et al., 2016; Dixon et al., 2019; Karnovsky et al., 2019; Fujitaka et al., 2020). Larger metatarsal adduction angles, smaller fourth-fifth intermetatarsal angles, and larger fifth metatarsal angles are linked to more frequent the fifth metatarsal stress fractures (Lee et al., 2013; O’Malley et al., 2016; Dixon et al., 2019; Karnovsky et al., 2019). These findings are consistent with biomechanical theories that highlight the impact of bone geometry on fracture susceptibility. Nevertheless, studies like Karnovsky et al. (2019) and Dixon et al. (2019) have high biases in outcome measurement, which could affect the reliability of these findings.

The variability in measuring metatarsal morphology across studies limits the ability to draw definitive conclusions. Standardized measurement protocols and larger sample sizes are needed to confirm these associations. The relationship between metatarsal morphology and stress fracture risk may be more complex than previously understood and requires a combination of anatomical structures and biomechanical properties of the foot.

### 4.2 Sports related

#### 4.2.1 Sports type

Fifth metatarsal stress fractures (MT5 SF) are prevalent among soccer players and are influenced by a complex interplay of factors. Notably, the anatomical structure of the foot plays a pivotal role, as does the repetitive stress and rapid changes in load experienced during the sport. The increased peak pressures observed in the non-dominant foot also suggest that asymmetrical loading and stress distribution may be significant contributors to the development of MT5 SF. Furthermore, the identification of these specific risk factors allows for a more targeted approach to prevention and intervention strategies.

In long-distance running, the prevalence of stress fractures among certain populations, such as college runners and ultramarathon participants, points to the inherent risks associated with endurance sports. The correlation between the popularity of running and the rise in running-related injuries (RRIs), as noted by Stolwijk et al. (2010) and Tenforde et al. (2013), highlights the importance of understanding the biomechanical and physiological demands of the sport. The recommendation for gradual mileage increases to mitigate the risk of RRI aligns with the broader preventative strategies that consider individual athlete characteristics, such as those proposed by Miller et al. (2019) and Azevedo et al. (2017).

These studies emphasize the significance of dynamic assessments in identifying athletes at risk, moving beyond static measurements to a more comprehensive understanding of an athlete’s biomechanical profile during movement. In summary, these provide a more profound understanding of the intricate relationship between sports activities, individual athlete factors, and the risk of metatarsal stress fractures.

#### 4.2.2 Exercise intensity and step rate

High-intensity activities (such as soccer and long-distance walking) and low step rates are correlated with increased fracture risk. This suggests that both the intensity and rhythm of physical activity are critical factors in fracture development. Regular exercise before training can reduce fracture risk during intense training periods, emphasizing the importance of conditioning and gradual intensity increases. Bias evaluation reveals that studies like Kliethermes et al. (2021) and Matsuda et al. (2017) have high biases in several domains, necessitating careful interpretation of their findings.

These findings emphasize the need for sport-specific injury prevention strategies. Coaches and healthcare providers should consider the unique demands of each sport and tailor their recommendations accordingly. Future research should investigate the interplay between sports-specific factors and individual anatomical variations to develop more effective prevention strategies.

#### 4.2.3 Sports fields and sports equipment

The mechanical environment, influenced by different types of sports fields and equipment, affects fracture risk. Extended play on artificial turf increases the risk of fifth metatarsal fractures (Miyamori et al., 2019). Footwear design, particularly stud configuration, impacts load distribution on the sole, raising fracture risk (Sun et al., 2017; Taylor et al., 2018; Lv et al., 2020). Minimalist shoes aim to reduce impact loads, but require an adaptation period to prevent stress fractures. More research is needed to design equipment that minimizes fracture risk. This underscores the need for proper adaptation and gradual transition in sports equipment to mitigate fracture risks.

### 4.3 Other factors

Young age has been identified as a significant risk factor for metatarsal stress fractures in certain sports (Ekstrand and Torstveit, 2012; Azevedo et al., 2017; Rice et al., 2019; Hotfiel et al., 2020). These findings suggests that younger athletes may have different bone properties and loading patterns, necessitating age-specific prevention strategies. Current research is limited in its ability to generalize findings across different age groups and sports. Future studies should focus on larger and more diverse populations to better understand the age-related risk factors for metatarsal stress fractures.

## 5 Limitation

There are several limitations in this review. Firstly, while this review provides a comprehensive analysis, the heterogeneity of the included studies limits the ability to perform a meta-analysis. Future research should aim to standardize definitions and methodologies to allow for more robust meta-analyses. Secondly, exploring the interplay of multiple risk factors in larger, more diverse populations will enhance our understanding of metatarsal stress fractures. Additionally, future investigations should prioritize incorporating more prospective studies. This approach can provide a comprehensive understanding of causal links between risk factors and fractures, informing injury prevention and management strategies in sports and healthcare.

## 6 Conclusion

In conclusion, our systematic review identifies key risk factors for metatarsal stress fractures in repetitive sports activities, emphasizing the complex interplay of anatomical, biomechanical, and sports-related influences. Relatively high arches and specific metatarsal morphologies are potentially significant anatomical risk factors. Biomechanically, foot inversion and eversion patterns are crucial, particularly among athletes, altering load distribution and increasing stress on specific metatarsals. Sports-related factors, including the type and intensity of activity, also play a critical role, with soccer linked to fifth metatarsal fractures, and long-distance running and recruit training associated with second or third metatarsal fractures.

Despite extensive research, study heterogeneity and inherent biases necessitate cautious interpretation. Standardized measurement protocols and longitudinal studies are needed to improve the robustness of future research. Understanding the unique demands of each sport and individual anatomical variations can inform personalized injury prevention strategies, tailored orthotics, specific training regimens, and careful management of training loads.

This review underscores the complexity of metatarsal stress fractures and highlights the need for comprehensive, multifactorial approaches to their prevention and management. By advancing our understanding of the underlying mechanisms, more effective strategies can be developed to reduce the incidence of these injuries and improve the health and performance of athletes.

## Data Availability

The original contributions presented in the study are included in the article/Supplementary Material, further inquiries can be directed to the corresponding authors.
