# Supplementary material for: Risk factors of metatarsal stress fracture associated with repetitive sports activities: a systematic review
Source: Front Bioeng Biotechnol. 2024 Aug 8;12:1435807. doi: 10.3389/fbioe.2024.1435807 (PMC11338896; doi:10.3389/fbioe.2024.1435807)
Supplement: Supplementary file 1 [file Table1.DOCX]

Supplementary Material

Risk Factors of Metatarsal Stress Fracture Associated with Repetitive Sports Activities

—— a Systematic Review

Sun JY^1,2,†^, Feng CL^2,†^, Liu YM^2^, Shan MJ^2^, Wang ZL^2^, Fu WJ^1,*^, and Niu WX^2,*^

*** Correspondence:** Corresponding Author: [niu@tongji.edu.cn](mailto:niu@tongji.edu.cn); fuweijie@sus.edu.cn

# Supplementary Figures and Tables

## Supplementary Tables

**Supplementary Table 2.** **Summary of the included literature**

| **Reference** | **Study type** | **Subject Info** | | | **Sports type** | **Injury type** | **Outcome** | | | **Evaluative/Experimental Method** | **Quality Assessment** |
| --- | --- | --- | --- | --- | --- | --- | --- | --- | --- | --- | --- |
|  |  | **Number** | **Age**  **(years)** | **BMI**  **(kg·m-2)** |  |  | **Anatomical environment of the foot** | **Sports related** | **Other factors** |  |  |
| Fujitaka et al. (2020) | Cohort Study | Total: 90 male university soccer players Case group: 30 Jones fracture Control group: 60 without Jones fracture | Case group: 19.6±0.7  Control group: 19.8±0.8 | Case group: 22.3±1.8 Control group: 22.5±2.3 | Soccer | Jones fracture | High medial longitudinal arch in Jones fracture group than control; The proximally longer MT5 in Jones fracture group than control | NA | NA | A mapping system to evaluate the radiographs | High |
| Hotfiel et al. (2020) | Cross-Sectional Study | 65 elite soccer players | Elite male soccer professionals: 23.4±4.0 Under-21 squad: 20.0±0.0 Under-17 squad: 16.0±1.0 Under-16 squad: 15.0±0.0 | - | Soccer | An increased PP under MT4&MT5 | NA | NA | Adolescents demonstrate asymmetric foot loading patterns with increased peak loads in the lateral aspects of the nonpreferred foot compared to adult elite soccer players | Sensor-loaded insoles | Medium |
| Lv et al. (2020) | Controlled Trial | 12 male football players  (knife stud vs triangle stud vs round stud) | 21.2±1.5yr | - | Soccer | MT5 SF | NA | Knife stud with high risk of MT5 SF; Round stud with high risk of medial and central MT SF | NA | The in-shoe plantar pressure measurement system | Medium |
| Karnovsky et al. (2019) | Case-Control Study | Total: 51 NFL players (96 feet) Case group: 15 Jones fracture feet Control group: 81 non-Jones fracture feet | 22.4±2.5 | - | Soccer | Jones fracture | Longer MT5; a large MT adduction angle; a smaller MT4-5 angle and a large MT5 angle in Jones fracture feet than control | NA | NA | Radiographic evaluation | High |
| Miyamori et al. (2019) | Cohort Study | Total: 1854 competitive-level football players Case group: 41 players with MT5 SF Control group: 1813 players without MT5 SF | 18.66±3.88 | 21.55±1.77 | Soccer | MT5 SF | NA | A more extended duration of sports activities on artificial turf with an increased risk of MT5 fractures | NA | Questionnaire | Medium |
| Miller et al. (2019) | Cohort Study | 37 professional football players with MT5 SF | 23±4.3 | - | Soccer | MT5 SF | NA | A return to sports at 8 weeks or less after SF increase time to union | NA | Radiographic evaluation | Medium |
| Kizaki et al. (2019) | Cross-Sectional Study | Total: 100 professional soccer players Case group: 15 soccer players Control group: 85 soccer players | Case group: 25±6 Control group: 25±5 | - | Soccer | MT5 SF | A wider malleolar slip angle | NA | NA | Radiographic evaluation | Medium |
| Saita et al. (2018) | Case-Control Study | Total: 60 male Japanese professional football players Case group: 20 players with a history of Jones fracture Control group: 40 players without a history of Jones fracture | Case group: 27.1±4.0 Control group: 26.2±4.3 | Case group: 22.9±0.9 Control group: 22.9±1.3 | Soccer | Jones fracture | Range limitation in hip internal rotation in the players with Jones fracture (p<0.05) | NA | NA | Injury history and physical examination | Medium |
| Taylor et al. (2018) | Controlled Trial | 20 competitive football players (standard shoe vs artificial turf-specific shoe) | 14.7±1.8 | - | Soccer | An increased MF in the central and lateral forefoot | NA | Cleat shoes with high plantar loading under the central and lateral forefoot | NA | The in-shoe pressure distribution measuring insole | Medium |
| Azevedo et al. (2017) | Controlled Trial | Total: 30 young adolescents Experimental group: 15 soccer players Control group: 15 non-soccer players | Experimental group: 14.0±0.7 Control group: 14.0±0.5 | - | Soccer | An increased PP under MT5 | NA | NA | Young soccer players with higher pressure observed in the nonpreferred foot | The plantar pressure mat system | High |
| Sun et al. (2017) | Controlled Trial | 16 male soccer players | 20.2±1.4 | - | Soccer | An increased PP &PTI in the medial and lateral forefoot | NA | Hard outsole with high PP and force-time integral in medial forefoot | NA | Lower limb kinematics and kinetics | Medium |
| Matsuda et al. (2017) | Case-Control Study | Total: 335 collegiate male soccer players Case group: 29 players with a history of MT5 SF Control group: 306 players without a history of MT5 SF | Case group: 20.0±1.1 Control group: 20.1±1.1 | - | Soccer | MT5 SF | High arch height (p>0.05); inverted forefoot and everted rearfoot alignments | Long-distance running and higher intensity activities (midfield position football athletes) | NA | Measurement of the foot length, arch height, weight-bearing & non-weight-bearing leg–heel alignment, forefoot angle relative to the rearfoot and foot pressure | High |
| Carl et al. (2014) | Controlled Trial | 17 elite male soccer professionals | 23.0±4.0 | - | Soccer | An increased PP under MT1 & MT4&MT5 | NA | Soccer boots generate excessive foot loadings predominantly under the lateral midffot, as compared with running shoes | NA | Plantar pressure measurements | Medium |
| Ekstrand et al. (2013) | Cohort Study | 64 male professional football teams | Total cohort: 25±5 MT5 SF: 23±3 | - | Soccer | MT5 SF | NA | At the beginning of the competition season, especially the preseason training period | Young soccer players with higher risk suffering MT5 SF | Follow-up report & Radiographic evaluation | High |
| Ekstrand et al. (2012) | Cohort Study | 54 football teams (2379 players) | UCL: 25.7±4.5 SWE: 24.8±4.7 ART: 25.0±4.8 | - | Soccer | MT5 SF | NA | At the beginning of the competition season, especially the preseason training period | Young soccer players with higher risk suffering MT5 SF | Follow-up report | Medium |
| Lee et al. (2011) | Case-Control Study | Total: 100 male soccer players Case group: 50 players with MT5 SF Control group: 50 players without MT5 SF | Case group: 19.5±2.4 Control group: 25.3±3.7 | - | Soccer | MT5 SF | Inverted rearfoot in players with MT5 SF (p<0.05); a smaller MT4-5 angle and a large MT5 angle in case group; a larger CP angle (high arch height) | NA | NA | Radiographic Measurements | Medium |
| Hetsroni et al. (2010) | Case-Control Study | Total: 20 professional soccer players Case group: 10 players with a unilateral SF of the proximal MT5 Control group: 10 players without SF | Case group: 24.0±2.6 Control group: 21.0±3.0 | Case group: 23.3±1.9 Control group: 24.0±1.5 | Soccer | MT5 SF | Non-significance in static Foot Measurements (p>0.05) | NA | NA | Static evaluation of foot structure & Dynamic evaluation | High |
| O'Malley et al. (2016) | Case-Control Study | Total: 10 NBA players with MT5 fracture Refracture group: 3 Control group: 7 | Refracture group: 24.0±1.7 Control group: 26.1±7.3 | Refracture group: 25.7±2.2  Control group: 25.7±1.7 | Basketball | Jones fracture | A large MT adduction angle; a smaller MT4-5 angle and a large MT5 angle in refracture group | NA | NA | Radiographic Measurements | Medium |
| Dixon et al. (2019) | Cohort Study | Total: 171 recruits Case group: 7 recruits with MT2 SF; 14 recruits with MT3 SF Control group: 150 recruits with no injury | MT2: 20.86±2.12 MT3: 19.86±2.60 Control group: 21.38±3.01 | - | Recruit training | MT2 & MT3 SF | A higher arch height in the MT2 SF group (p<0.05); Lower foot abduction in the MT2 SF group (p<0.05) | NA | Younger age in the MT3 SF group (p<0.05) | Passive range of ankle dorsi-flexion, dynamic peak ankle dorsi-flexion and plantar pressures during barefoot running | Medium |
| Rice et al. (2019) | Controlled Trial | 32 male Royal Marines recruits | 22.9±3.84 | 25.9±2.0 | Recruit training | The reduction in loading under MT1-2 | NA | The reduction in loading under MT1-2 after long-period training activity | NA | Measurement of plantar pressure variables and ankle dorsiflexion | High |
| Pihlajamäki et al. (2019) | Cohort Study | Total: 4029 men performing military service Case group: 3985 men with SF Control group: 44 men without SF | Case group: 19.2±0.6 Control group: 19.2±1.1 | Case group: 22.7±3.9 Control group: 23.3±3.8 | Recruit training | MT SF | NA | Small number of fatigue fractures in MT with regular and repeated high-intensity physical activities before | NA | Questionnaire | Medium |
| Shaffer et al. (2006) | Cohort Study | 2962 female Marine Corps recruits | 19.2±2.1 | 21.6±1.9 | Recruit training | MT SF | NA | Small number of MT SF with regular and repeated high-intensity physical activities before | NA | Anthropometric & Questionnaire& Aerobic Fitness Measurements | Medium |
| Kliethermes et al. (2021) | Cohort Study | 54 healthy collegiate cross country runners (21 men, 33 women） | 19.5±1.3 | 20.3±1.7 | Cross country running | MT SF | NA | Low step rate during running at a self-selected moderate intensity speed with BSI | NA | Collection of whole-body kinematics, ground reaction forces and BMD measures | High |
| Bergstra et al. (2015) | Controlled Trial | 18 female endurance runners | 23.6±3 | - | Running | An increased PP, MP and PTI in the medial forefoot, central forefoot, and lateral forefoot | NA | Too fast switch to minimalist shoes with high risk of MT SF | NA | In-shoe plantar pressure measurements | High |
| Wellenkotter et al. (2014) | Controlled Trial | 38 healthy recreational runners (19 men, 19 women) | 23.0±3.5 | - | Running | An increased CT, FTI and PTI in MT region | NA | Increased running cadence with high CT, FTI and PTI in MT region | NA | The Pedar-X insole shoe system | Medium |
| Tenforde et al. (2013) | Cohort Study | Total: 748 high school runners Girls: 442 (23 SF, 405 non-fractures) Boys: 306 (11 SF, 262 non-fractures) | Girls with SF: 15.6±1.2 Girls without SF: 15.3±1.1 Boys with SF: 15.5±1.0 Boys without SF: 15.4±1.2 | Girls with SF: 18.7±1.5 Girls without SF: 20.0±2.5 Boys with SF: 19.5±1.8 Boys without SF: 20.0±2.8 | Running | MT SF | NA | NA | Prior fractures as a risk factor with both young female and male；girls with low BMI and late menarche；boys with prior participation have low risk of SF | Follow-up questionnaire report | Medium |
| Bischof et al. (2010) | Case-Control Study | Total: 24 females Case group: 9 females with a history of MT2/3 SF Control group: 15 females without a history of MT SF | Case group: 24.4±6.24 Control group: 22.07±3.41 | - | Running | MT2 & MT3 SF | NA | Over training | NA | Kinematic and kinetic analysis | High |
| Stolwijk et al. (2010) | Controlled Trial | 62 (30 men, 32 women) | Men: 43.8±8.0 Women: 44.8±5.3 | Men: 26.6±3.4 Women: 24.4±3.4 | Long- distance walking | An increased MP, PP and PTI under MT4-5 head | NA | MP, PP, and PTI increased significantly under MT4-5 head | NA | Plantar pressure measurements | Medium |
| Nagel et al. (2008) | Controlled Trial | 200 marathon runner (167 men, 33 women) | 39.5±8.8 | 23.15±2.0 | Marathon running | An increased PP under MT2 & MT3 | NA | The increased peak pressure under MT heads after long distance running | NA | Plantar pressure measurements | High |
| Weist et al. (2004) | Cohort Study | 30 experienced runners | 34.5±8.8 | 22.0±2.0 | Running | An increased PF, PP and impulse under MT2 & MT3 | Muscle fatigue | NA | NA | Plantar pressure measurements | High |
| Bennell et al. (1996) | Cohort Study | Total: 111 competitive track and field athletes (53 women, 58 men) | Men: 20.3±2.0 Women: 20.5±2.2 | Men: 21.8±1.7 Women: 21.1±1.8 | Track and field | MT SF | NA | Sprinters, hurdlers, jumpers, and multievent athletes sustained more MT SF | Women with more MT SF | Questionnaire | Medium |
| Sullivan et al. (1984) | Cohort Study | 51 runners (36men, 15women) | Men: mean 18.2  Women: mean 23.7 | - | Running | MT SF | Pes planus with SF | Weekly training distances greater than 20 miles, hard training surfaces, and training regimen modification | NA | Questionnaire & Radiographic evaluation | Medium |

MT= Metatarsal, SF= Stress Fracture, BMD= Bone Mineral Density, NA= Not Available, MF= Maximum Force, MP= Mean Pressure, PP= Peak Pressure, PTI= Pressure Time Integral, CT= Contact Time, PF= Peak Force, FTI= Force Time Integral, CP= Calcaneal Pitch, UCL= UEFA Champions League, SWE= Swedish Super-League, ART= UEFA Artificial Turf.
